# Inducible nitric oxide synthase blockade with aminoguanidine, protects mice infected with *Nocardia brasiliensis* from actinomycetoma development

**DOI:** 10.1371/journal.pntd.0008775

**Published:** 2020-10-22

**Authors:** Mario C. Salinas-Carmona, Ossian Longoria-Lozano, Humberto R. Garza-Esquivel, Juan López-Ulloa, Jorge Reyes-Carrillo, Anna Velia Vázquez-Marmolejo

**Affiliations:** Universidad Autonoma de Nuevo Leon, Facultad de Medicina y Hospital Universitario, Servicio de Inmunología, Monterrey, Nuevo Leon, Mexico; University of Tennessee, UNITED STATES

## Abstract

Mycetoma is a chronic infectious disease that can be caused by fungi or bacteria, *Madurella mycetomatis and Nocardia brasiliensis* are frequent etiologic agents of this disease. Mycetoma produced by bacteria is known as actinomycetoma. In mycetoma produced by fungi (eumycetoma) and actinomycetoma, diagnosis of the disease is based on clinical findings: severe inflammation, with deformities of affected tissues, abscesses, fistulae, sinuses and discharge of purulent material that contains micro colonies of the etiologic agent. Microscopic examination of infected tissue is similar regardless of the offending microbe; hallmark of infected tissue is severe inflammation with abundant neutrophils around micro colonies and granuloma formation with macrophages, lymphocytes, dendritic and foamy cells. Even though medical treatment is available for mycetoma patients, amputation, or surgical intervention is frequently needed. The pathogenesis of actinomycetoma is little known, most information was obtained from experimental animal models infected with bacteria. In other experimental mice infections with different microbes, it was demonstrated that nitric oxide is responsible for the intracellular killing of *Mycobacterium tuberculosis* by activated macrophages. Nitric oxide is a free radical with potent stimulatory and suppressive effects in innate and adaptive immunity. The unstable nitric oxide molecule is produced by action of nitric oxide synthases on L-arginine. There are three nitric oxide synthases expressed in different cells and tissues, two are constitutively expressed one in neurons, and the other in endothelial cells and one that is inducible in macrophages. Aminoguanidine is a competitive inhibitor of inducible nitric oxide synthase. Its administration in experimental animals may favor or harm them. We used aminoguanidine in mice infected with *Nocardia brasiliensis*, and demonstrated that all treated animals were protected from actinomycetoma development. Anti *N*. *brasiliensis* antibodies and T cell proliferation were not affected, but inflammation was reduced.

## Author summary

Mycetoma is a chronic human infection, recently listed as a neglected tropical disease. Its diagnosis, treatment and prevention are complicated. In theory, infectious disease produced by bacteria or fungi may be preventable through vaccination; however, there is no available vaccine for this and other infections. This chronic infection usually affects the lower extremities and produce severe inflammation with deformities and disability. Current medical treatment is expensive and requires months or years to complete, and surgical intervention including amputation is frequent. Microbes that produce mycetoma live in soil, and enter the skin by accidental traumatic inoculation with spines. There is an urgent need to generate scientific information to identify possible therapeutic targets and vaccine development for treatment or prevention. An actinomycetoma model was created using mice under experimental conditions, to understand how these microbes produces damage and to comprehend the host response to clear the infection. In this work, aminoguanidine was given to experimental mice and it was found that this treatment, completely prevented inflammation and the mycetoma lesion. This information will be useful for the design of treatments that target not only the offending microbe but also to modulate the host immune response.

## Introduction

Mycetoma is a chronic human infection produced by fungi (eumycetoma) or bacteria (actinomycetoma). This disease causes a deforming inflammation of affected tissue, usually the extremities, and had been recently listed as a Neglected Tropical Disease [1].

Actinomycetoma studies both in human and mice demonstrate that active disease is characterized by an enhanced systemic anti *Nocardia brasiliensis* antibody response, simultaneously coexisting with an immunosuppressive local microenvironment [2–3].

We created an experimental actinomycetoma model in mice, with *Nocardia brasiliensis* ATCC700358 (formerly HUJEG-1) injected in the rear footpad, to study the host-parasite relationship [4]. This model has been useful to investigate the pathogenesis of actinomycetoma, since it reproduces the same macroscopic changes such as intense swelling with severe deformation of affected extremities. In human natural infection and experimental mice, clinical findings include abscesses ulcers, and sinuses that discharge micro colonies of the offending microbe known as granules. The hallmark of infected tissue in human and experimental mice is acute and chronic inflammation characterized by a large number of micro abscesses and granulomas, with foamy cells, macrophages, lymphocytes, and extensive fibrosis around the micro colonies of the bacteria or fungi.

The role of the immune response to clear intracellular bacterial infections produced by *Mycobacterium tuberculosis*, *Listeria monocytogenes* and *N*. *brasiliensis* is not clear; it has been a paradigm that the cellular immune response mediated by T lymphocytes is responsible for protection against these microbes and that the antibody response does not provide protection. The role of antibodies in clearing intracellular infections has been the subject of experimentation for the last 50 years [5]. However, controversy seems to diminish with the use of homogenous antibody preparations for passive immunity experiments specially with monoclonal antibodies. Recently it was found that IgM but not IgG anti-*N*. *brasiliensis* protects mice from developing actinomycetoma in experimental BALB/c mice in agreement with other authors that antibodies can control infection caused by an intracellular bacteria such as *N*. *brasiliensis* [6–8].

Nitric oxide (NO)was known to have antimicrobial properties before its imunoregulatory activities were recognized. This molecule, is produced by the reaction of the nitric oxide synthases and the aminoacid substrate L-arginine, to form L-citrulline and NO. The enzyme nitric oxide synthase is coded by one of three genes, two of which are constitutively expressed in neurons (nNOS), and endothelial cells (eNOS), and one gene is inducible in activated macrophages, neutrophils, dendritic and T cells (iNOS). This inducible form is known as nitric oxide synthase 2 (NOS2).

NO is an unstable free radical with a short life time of only seconds, but it can easily traverse biological membranes and affect pathogens viability by altering molecules such as DNA and proteins. NO is finally oxidized to nitrites and nitrates.

The NO molecule, is part of the reactive nitrogen species (NRS) similar to reactive oxygen species (ROS), these free radicals have been characterized as the ones responsible for the intracellular killing of bacteria by activated macrophages [9].

Small amounts of NO are constitutively produced and participates in important physiological events such as blood flow and blood pressure regulation; the expression of iNOS is extensively controlled at transcription, translational and posttranslational levels. On the other hand, iNOS is stimulated in macrophages by LPS, TNF-α and IFN-γ to locally produce high concentrations of NO. High NO concentrations, may contribute to tissue damage in chronic inflammation conditions [10].

It is now recognized that NO, plays an important role in adaptive and innate immunity because of its stimulatory and immunosuppressive properties. Local tissue conditions including oxygen and some cytokines concentration are known to determine which and how much NO activity will be present. Overproduction of this toxic molecule may be responsible for microbicide, cytotoxic and mutagenesis effects in chronic inflammatory processes [11–12].

Aminoguanidine (AG) is a potent selective inhibitor of inducible NOS.Its inhibition mechanism of action is competitive at substrate level, but also at the gene expression level (transcription, translation and posttranslational) [12].

AG was investigated because of its potent effect on diabetes animal models. In those models an anti-angiogenic effect was demonstrated, because it prevented the formation of advanced glycation of end products(AGES) [13].

A protective effect of AG administration, has been demonstrated in rats due to a decrease in peroxynitrite formation (ONOO). In rodents, it was found that an AG derived compound decreased proinflammatory cytokines and increase Il-10 [14].

AG administration to mice infected with *M*. *tuberculosis*, induced reactivation of latent infection with robust granuloma formation, and increase in bacillary load. This effect of AG treatment was not due to T cell suppression [15].

Contradictory findings of AG administration in mice show that in some infections, this treatment worsen infections, but using other bacteria, AG help to clear infection [16–17].

In the present work, we investigated the effect of AG administration in mice infected with *Nocardia brasiliensis*. Inflammation, IgG, IgM Anti-*N*. *brasiliensis* antibodies and T lymphocytes were determined during the acute and chronic phases of experimental infection. AG did not affect *Nocardia brasiliensis* viability and prevented acute and chronic inflammation. Unexpectedly treated animals cleared the infection and completely prevented actinomycetoma development.

## Materials and methods

### Ethics statement

Animals were cared and handled according to International Review Board Regulations and the Mexican Animal Protection Law NOM-062-ZOO-1999. The study was approved by the Bioethics Committee of the School of Medicine, Universidad Autonoma de Nuevo León (Approval number IN09-002).

### Mice

Female BALB/c mice, 12- to 14-weeks old were used in these experiments. Mice received rodent food and water *ad libitum*.

### *Nocardia brasiliensis* strain ATCC 700358

The bacterial cell suspension was prepared as previously published [4], briefly: *N*. *brasiliensis* (ATCC 700358) was cultured at 37°C in brain heart infusion broth (BHI) (Difco, BD) for 72 h to rich the log growth phase. To obtain a single cell suspension, the biomass was collected and washed with sterile saline solution, bacterial clumps were dissociated using a homogenizer. After another wash step, the residual cell clumps were eliminated by centrifugation at 1600 rpm for 5 minutes. The supernatants containing the unicellular cells in suspension were collected and adjusted to 1 x 10^6^ bacterial cells in 0.1 ml of saline solution.

### *N*. *brasiliensis* viability in the presence of AG

We used a 96 well flat bottom tissue culture plate with 100 μl of a 4% AG solution. From well A 1 to 12, two-fold serial dilution was prepared. *N*. *brasiliensis* ATCC 700358 in 100 μl of BHI culture media containing 1x10^6^ CFU was added to each well. *N*. *brasiliensis* amikacin sensitivity was tested in wells B 1 to 3 at a concentration of 0.25 μg/ml. *N*. *brasiliensis* control growth in BHI was included in wells B 7 to 9.

### Survival rate of mice infected and treated with AG

Female BALB/c mice, 12 week-old, were used in four different groups with 15 animals in the first group and 10 mice in each of the other three groups; The first group of 15 animals received a 2% AG in water *ad libitum* and were infected with the bacteria. The second group was infected but did not receive AG in water. The third group received AG in water but were not infected. The last group was a control that did not receive AG and was not infected. The mice that were infected with *N*. *brasiliensis* in one rear foot pad, were injected with 100 μl of a solution containing 1 x10^6^ CFU of the bacteria in the log growing phase. The animals included in this experiment were only used to determine the survival rate.

### Inflammation in mice infected and treated with AG

Female BALB/c mice, 12 weeks old were used in four different groups with 25 animals each group. The first group received 2% AG in water *ad libitum* and were infected with the bacteria. The second group was infected but did not received AG in water. The third group received AG in water but were not infected. The last group was a control that did not receive AG and was not infected. The mice that were infected with *N*. *brasiliensis* in one rear foot pad, were injected with 100 μl of a solution containing 1 x10^6^ CFU of the bacteria in the log growing phase and were euthanized by cervical dislocation at different times. After being euthanized, blood samples and peritoneal macrophages were obtained, spleen and infected foot pad were aseptically removed in 5 animals of each group at 0, 7, 30 and 60 days post infection. Footpads from animals were fixed in 5% formalin solution and processed for histopathology to obtain tissue sections with hematoxylin and eosin for microscopic study. Inflammation is shown in cubic centimeters using the ellipsoid formula. (4/3π*abc*).

### iNOS blockade with AG demonstrated by nitrates and nitrites quantification in plasma

After being euthanized by cervical dislocation, plasma from heparinized blood was obtained at day 60 of the experiment by cardiac puncture to indirectly measure the NO concentration to demonstrate that nitric oxide synthase was blocked during AG treatment. Nitrate and nitrite were quantified in plasma using colorimetric Calbiochem Nitric Oxide Assay.

### iNOS blockade with AG effects on macrophage killing ability against *N*. *brasiliensis*

At day 60, AG treated non infected and non treated non infected mice were euthanized by cervical dislocation to obtain peritoneal macrophages briefly described here; 5 ml of cold, nitrate free DMEM medium was injected into the peritoneal cavity of naïve donor animals. Cell suspensions from the peritoneal cavities from both groups, were incubated for 2 hours at 37° C. in tissue culture 6-well plates; non-adherent cells were discarded by eliminating the supernatant 3 times with phosphate-buffered saline solution (PBS). Adherent cells were removed using a scraper and centrifuged at 1600 rpm for 5 minutes and the supernatant was discarded, pelleted cells were suspended in DMEM culture media and adjusted to 3x10^5^ cells per well. A suspension of *N*. *brasiliensis* opsonized for 30 minutes with hyperimmune sera from infected mice with actinomycetoma, was added at MOI 3:1 (3 bacterial cells per 1 macrophage). Adherent infected cells with the opsonized bacteria were cultured in 24-well tissue culture plates at 37° C with 5% CO_2_ for 24, 48 and 72 hours. At the end of culture, the supernatant was eliminated, adherent cells were lyzed with cold distilled water and vigorous pipetting; 100 μl samples from the obtained supernatant were incubated in blood agar Petri dishes to determine colony forming units of *N*. *brasiliensis* released from lyzed macrophages after 7 days of culture at 37° C.

### Anti *Nocardia brasiliensis* antibody determination

Mice sera from AG treated and non treated groups, were obtained 7 and 45 days after infection to investigate IgM and IgG Anti-*N*. *brasiliensis* antibodies using an ELISA technique standardized in our lab [18].

As previously published, purified immunodominant 26- and 24-kDa proteins were obtained from *N*. *brasiliensis* batch culture to setup an ELISA, which is briefly described here as follows: 0.5 μg of the purified protein per well, were incubated in 96 wells polystyrene plates, after three washes with PBS-Tween 20, plates were blocked with 5% skim milk and washed five times. Diluted sera 200 μl of from experimental and control mice were incubated for 1 h at 37° C and washed again. An anti-mouse IgG peroxidase conjugate was added and incubated for 1 hr. A chromogen substrate composed of o-phenylendiamine and hydrogen peroxide was added in addition to sulfuric acid to stop the reaction. Absorbance was read with a semiautomatic plate reader at 492nm. Results are presented as absorbance values.

The AG treated and infected, and the controls study group were immunized simultaneously with 0.1ml of a 2% sheep red blood cells (SRBC) suspension in the peritoneal cavity as non related N. *brasiliensis* antigen. Animals were euthanized 4 days after immunization and spleen cells were obtained and tested for IgM plaque forming cells (IgM-PFC) according to the Cunningham PFC technique [19].

### Lymphocyte proliferation assay

Mice from infected and AG treated and non treated groups were euthanized; their spleens were aseptically removed and mechanically dissociated to prepare a mononuclear cell suspension at a concentration of 10^6^ per ml in RPMI 1640 culture media supplemented with 2mM glutamine, 25 mM HEPES, 100 U of penicillin, and 10% heat inactivated bovine serum. Sterile flat bottom, 96-well tissue culture plates (Falcon 3020) were used to incubate 2x10^5^ mononuclear cells suspended in 0.2 ml of supplemented medium. Triplicate cultures were stimulated with 0.1 μg/μL phytohemagglutinin (PHA) and incubated at 37°C in5% CO_2_ for 5 days. Tritiated thymidine (37kBq-well: Amersham) was added 16h before harvesting with a semiautomatic micro-harvester (MASH II). Thymidine incorporation was quantified with a liquid scintillation counter (Beckman). Counts per minute (CPM) results are the mean of triplicate cultures. Data was used to calculate the stimulation index.

### Histopathological study

After euthanasia, the foot pads from infected mice were aseptically removed, tissues were fixed in 5% formalin solution and processed for hematoxylin and eosin (H&E) staining.

### *Nocardia brasiliensis* bacterial load after AG treatment

Euthanized mice at the end of the experiment after AG treatment and infection, were used to quantitate bacterial load in blood, liver, spleen and kidney. The tissues were homogenized to prepare cell suspensions, and 20 μl samples were taken and cultured in Petri dishes with blood agar at 37°C for 7 days. Colony forming units were quantitated.

### Statistical analysis

Mean, standard deviation, ANOVA, Kruskal Wallis, Bonferroni and the t-Test were used to calculate the statistical significance of results as shown in each figure legend.

## Results

### *N*. *brasiliensis* viability is not affected by different concentrations of AG

**Fig 1:**
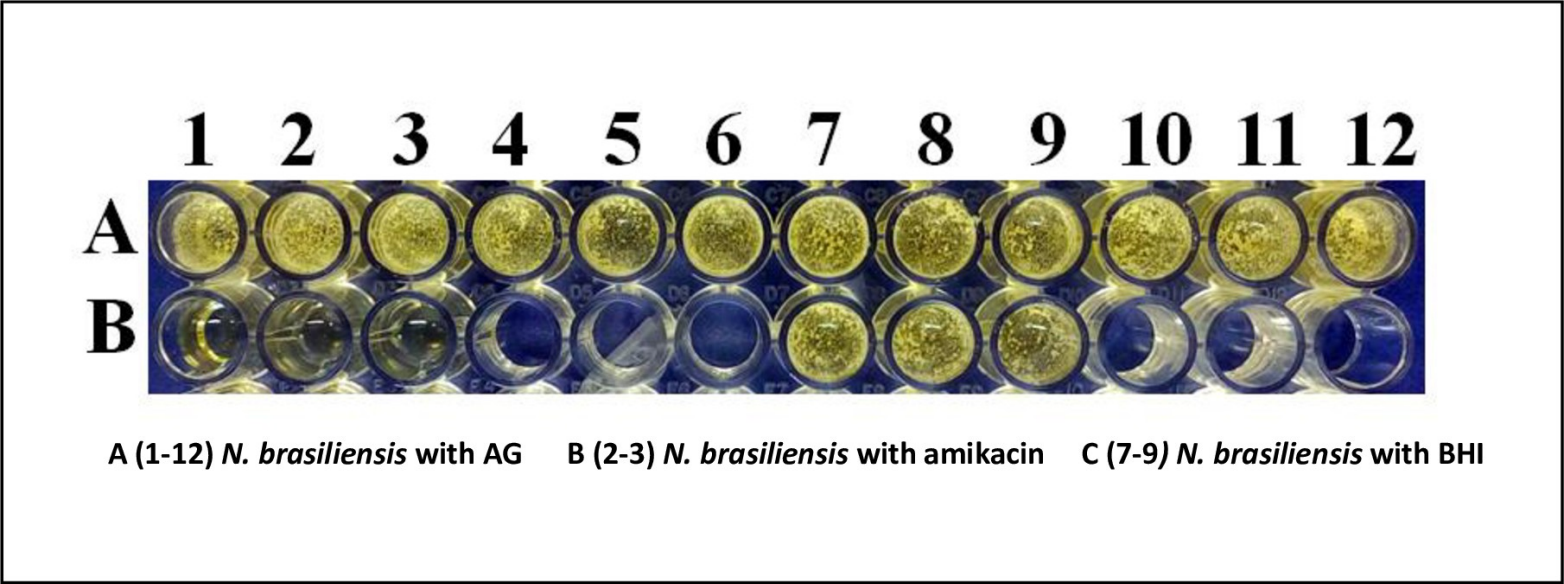
*N brasiliensis* viability is not affected by AG at different concentrations. Lane A wells 1 to 12 show *N brasiliensis* in BHI culture media with different AG concentrations. Lane B wells 1 to 3 demonstrate *N*. *brasiliensis* sensitivity to amikacin with no colony survival after 72 h of culture. Wells B 7 to 9 contain *N*. *brasiliensis* in BHI media.

*N*. *brasiliensis* cultured with AG in BHI medium showed that AG has no deleterious effect on bacterial growth, viability and the morphology of colonies. AG even at high concentrations did have not an effect compared to amikacin incubation in cultures at 37° C for 3 days, as shown in Fig 1.

### The survival rate of mice infected and treated with AG

**Fig 2:**
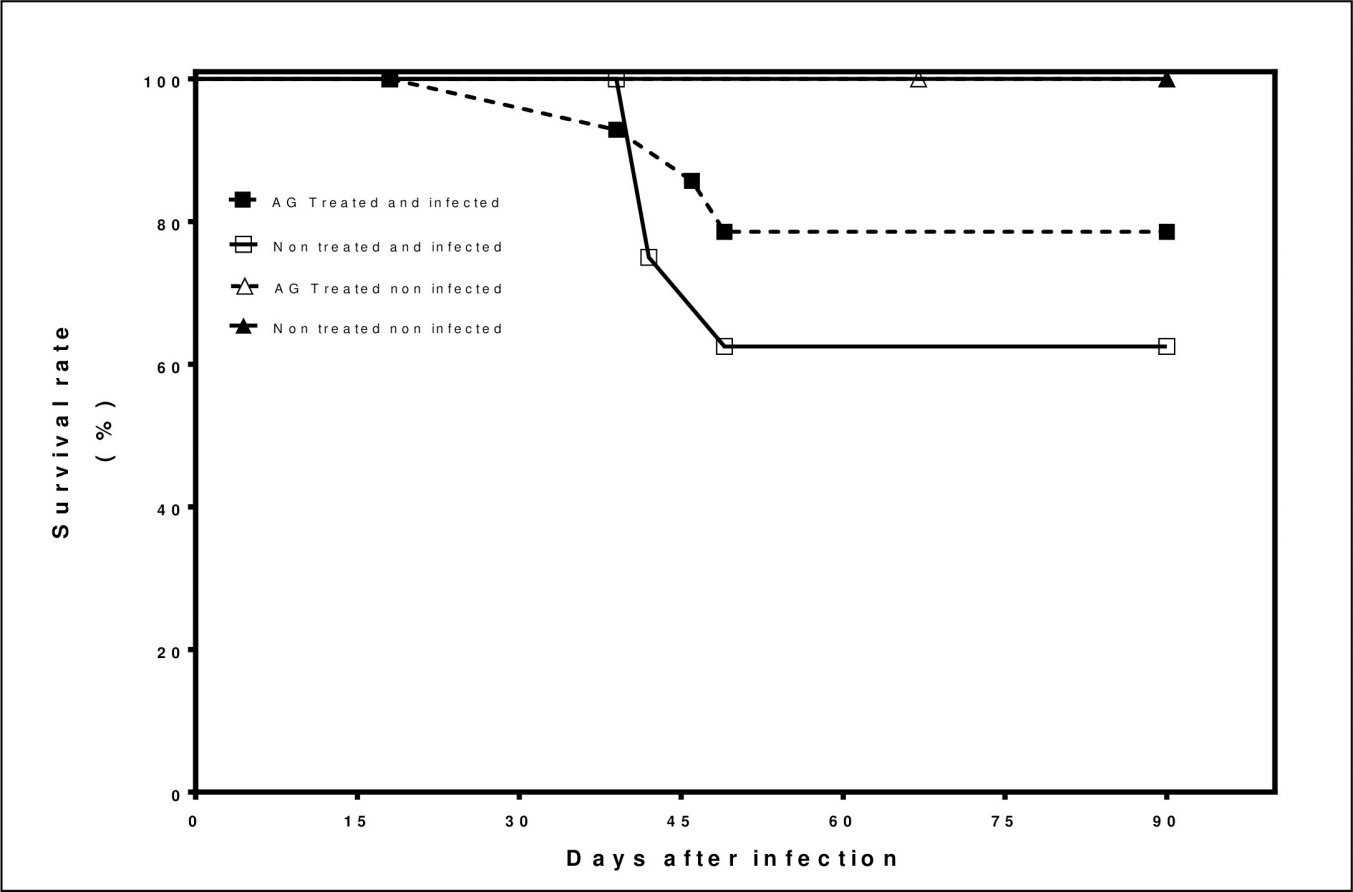
The survival rate of BALB/c mice infected with *Nocardia brasiliensis* and treated with AG. Results represent the percentage of living animals up to 90 days of infection with and without AG and infected and non infected. Typical results of 1 experiment with 45 animals.

The survival rate is presented as a percentage of living mice at different times, up to 90 days. In the first group of 15 animals infected with *N*. *brasiliensis* that received AG treatment, 78.5%. survived. In the second group non-treated and infected, 62.5%. survived In the third group that received AG treatment and non-infected 100% survived at the end of the experiment. The fourth group non-infected non-treated100% survived. The AG treated group and infected had less mortality compared to untreated infected group as shown in Fig 2.

### Inflammation in mice infected and treated with AG

**Fig 3:**
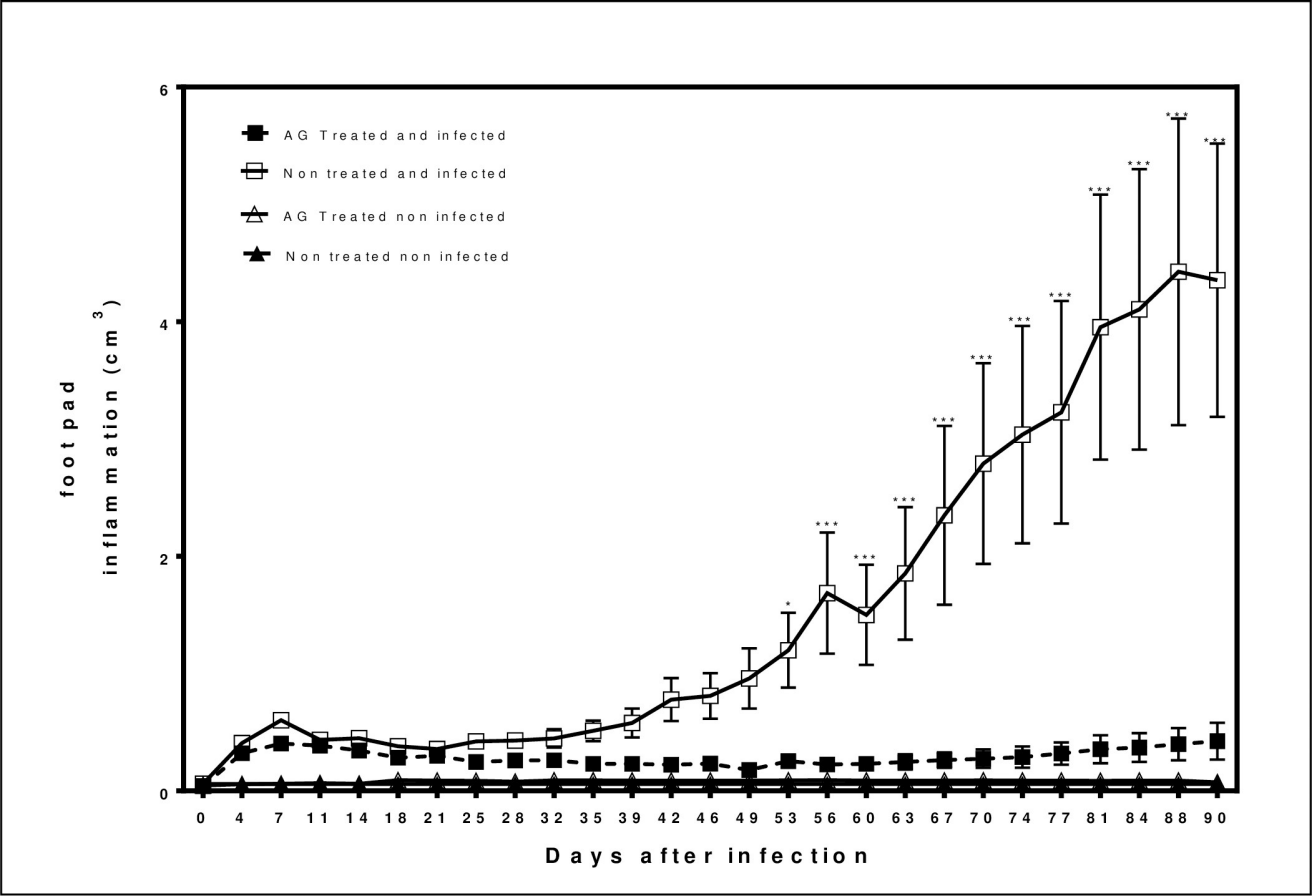
Inflammation in mice infected and treated with AG. Results of footpad inflammation obtained with a caliper analyzed with a 2-way ANOVA with repeated measures with Bonferroni posttest. (*p<0.05, *** p< 0.001).

Clinical evolution was followed for 90 days for the four groups: AG treated and infected, non-treated and infected, and the control groups that received AG but non-infected, and the fourth group that did not receive AG and was non-infected. At day 7 after infection, acute inflammation determined as the volume of inflammation, there is no difference in volume between the treated and untreated groups. However, in the chronic phase of the actinomycetoma, on days 30, 60 and 90 after the experimental infection, the AG treated group showed no signs of inflammation compared to the infected but untreated group as summarized in Fig 3.

**Fig 4:**
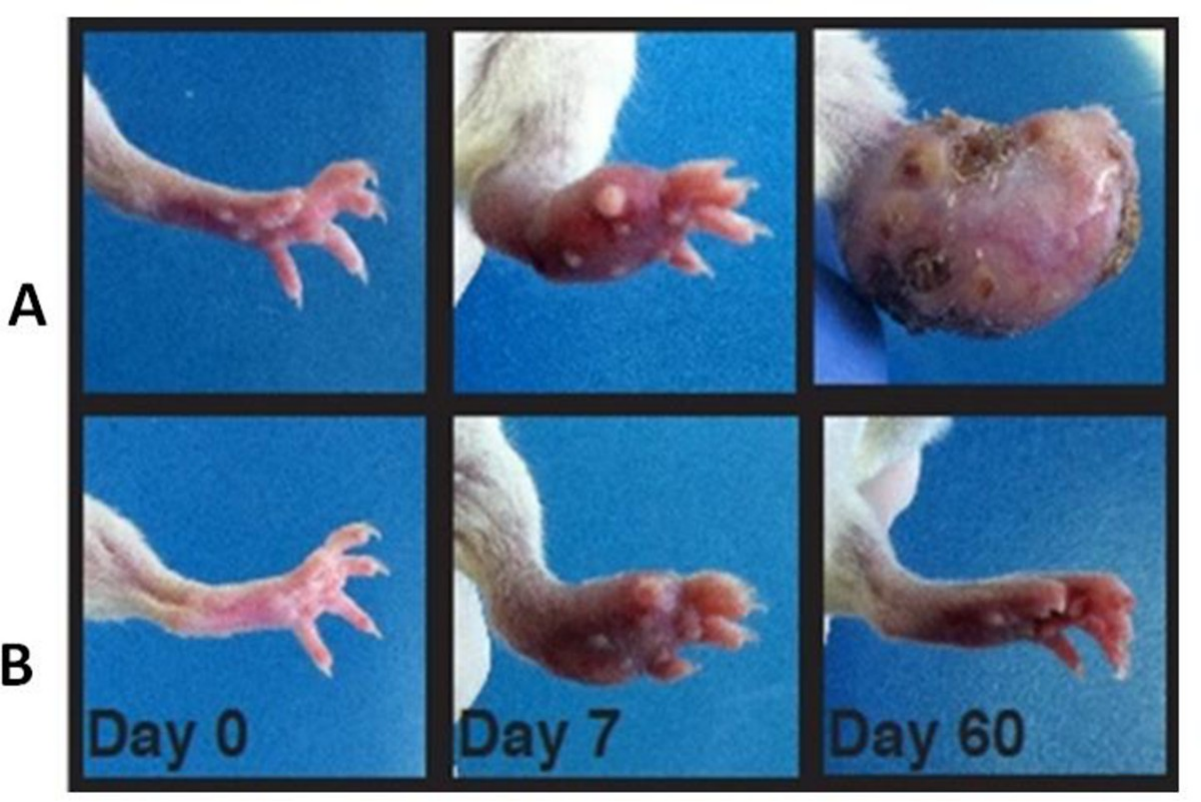
AG treatment prevents actinomycetoma. Clinical aspect of rear foot pads from mice infected with *N*. *brasiliensis*. A) Non-treated and infected B) AG treated and infected.

AG treatment completely prevented actinomycetoma development; treated animals lacked inflammation, ulcers, abscesses and deformities in the infected site, in comparison with the non treated and infected group which developed full blown mycetoma as shown in Fig 4.

### iNOS was blocked with AG treatment

**Fig 5:**
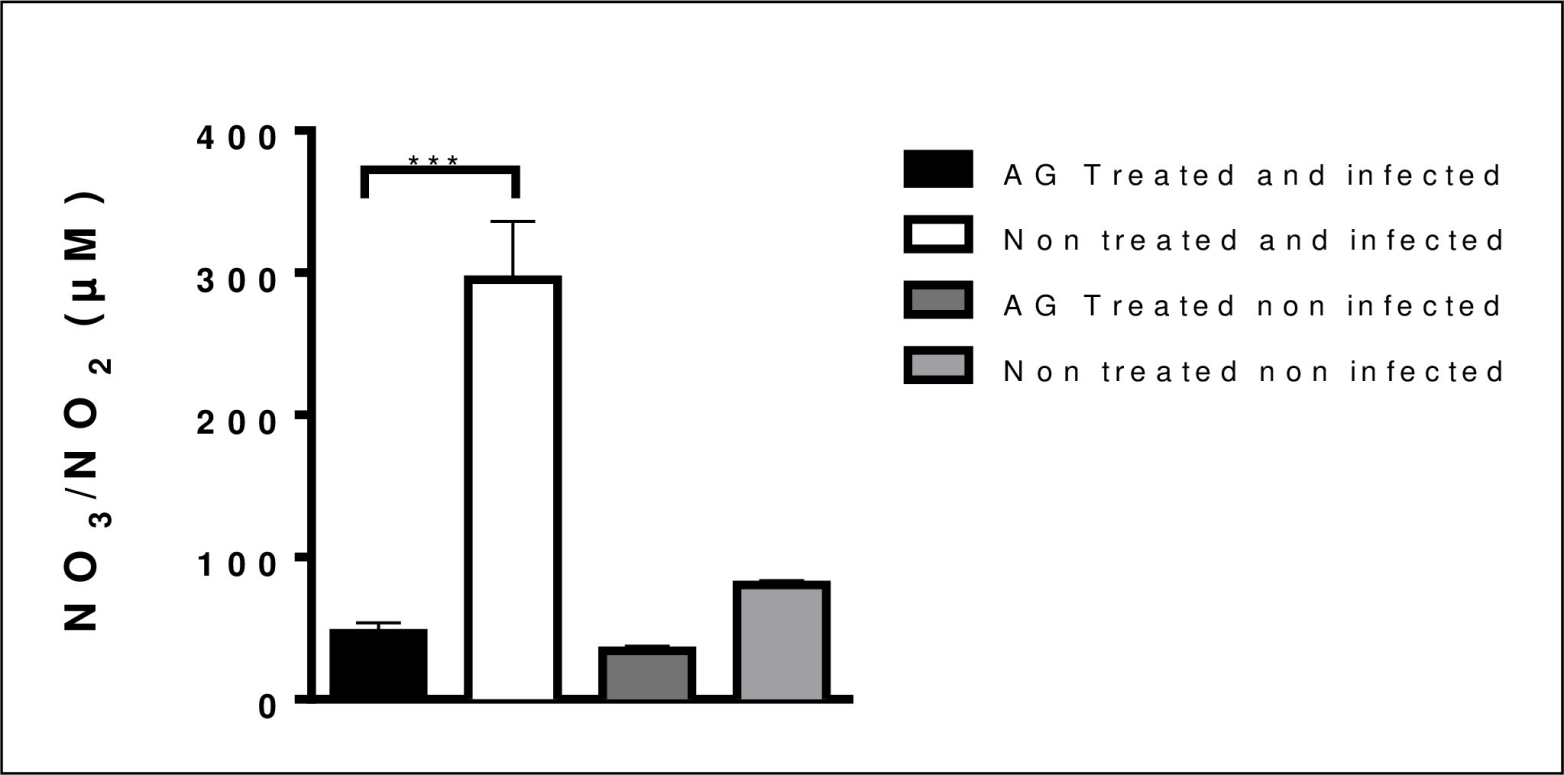
Nitric oxide concentration in mice treated with AG is decreased in plasma. Inducible nitric oxide synthase (iNOS) inhibition was decreased and proves that iNOS inhibition is effective. (***p<0.001).

To asses that iNOS was blocked by AG treatment, mice infected with *N*. *brasiliensis* were used to quantitate nitrate and nitrite formation in plasma, results showed that indeed iNOS activity was decreased, compared with infected untreated group as presented in Fig 5.

### Macrophage intracellular killing of bacteria is decreased by AG treatment

**Fig 6:**
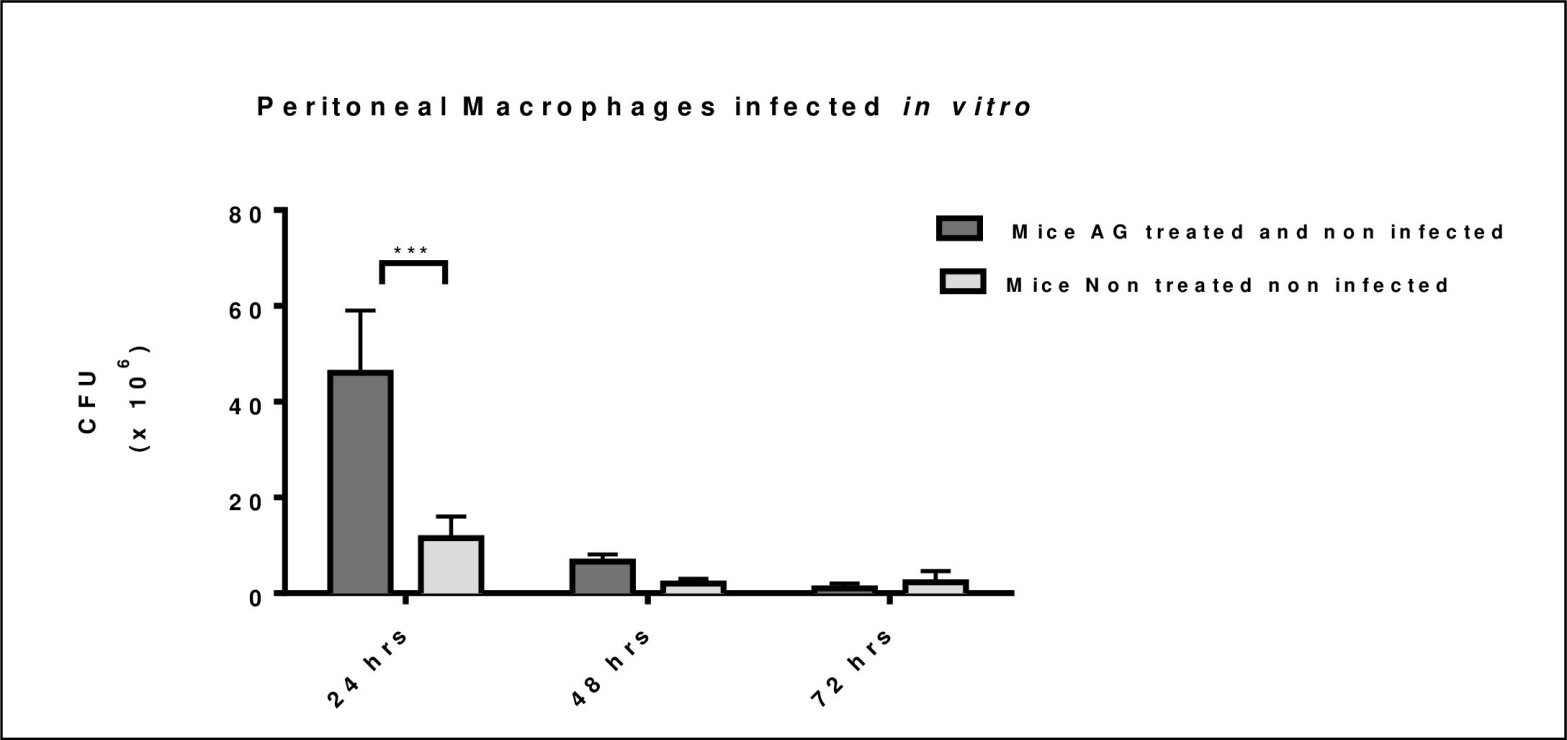
AG treatment decreased macrophage ability to kill *N*. *brasiliensis* in vitro at 24 hours. Macrophages from AG treated and non-infected mice decreased intracellular killing after 24 hours of in vitro infection. Intracellular killing was recovered at 48 and 72 hours after in vitro infection. (***p <0.001).

Macrophages from naïve mice treated for 60 days with AG and non-infected decreased their ability to kill intracellular bacteria at 24 hours after in vitro infection as presented in Fig 6. This killing effect disappeared 48 and 72 hours after in vitro infection, when macrophages were able to kill intracellular *N*. *brasiliensis*.

### IgG and IgM Anti *N*. *brasiliensis* antibody response in treated and infected mice

**Fig 7:**
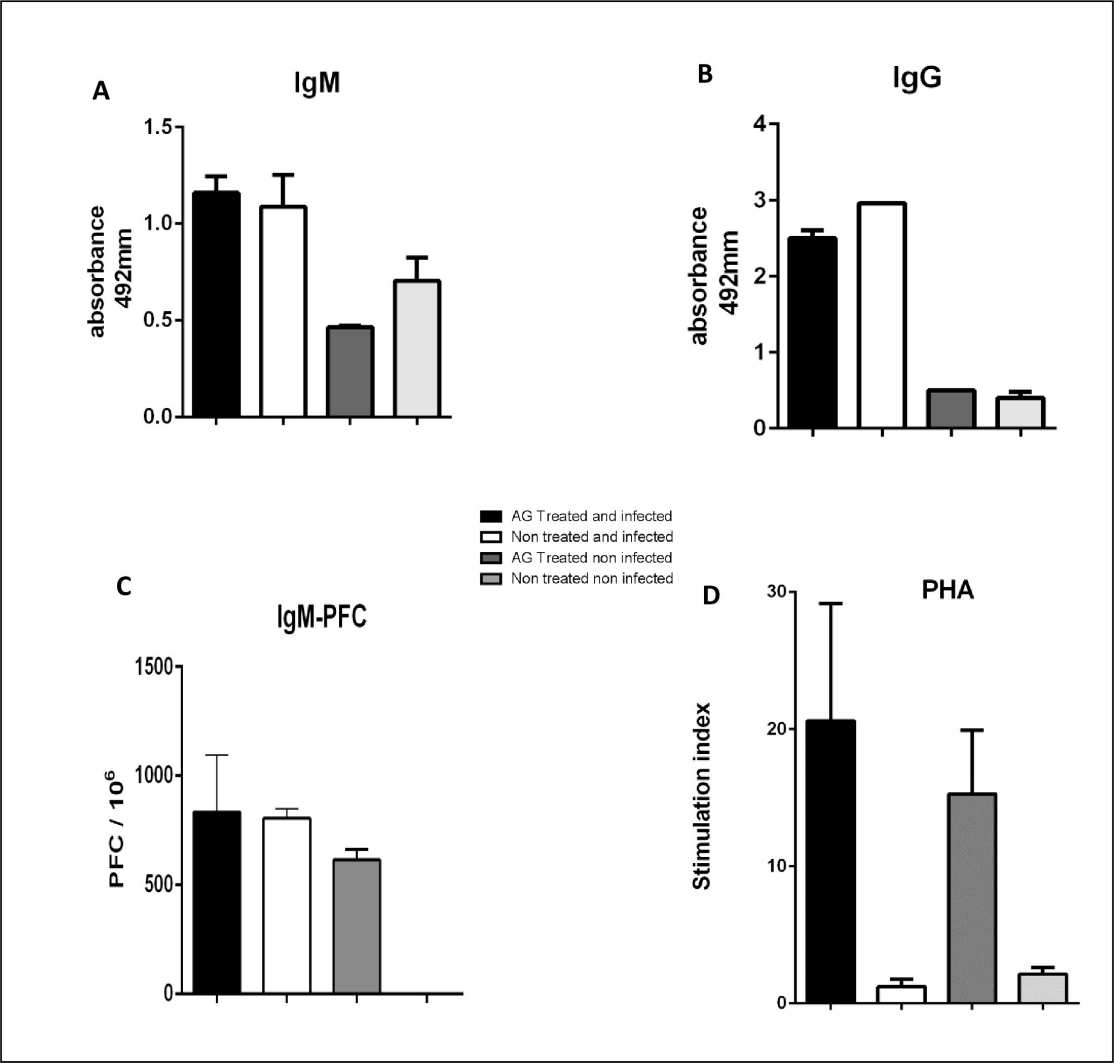
IgG and IgM anti *N*. *brasiliensis* antibodies is not affected by AG treatment. IgM and IgG antibody titer was determined with an ELISA test (A, B) (p< 0.005 and p< 0.001 respectively). IgM-PFC with SRBC as unrelated antigen was not affected with AG treatment either (C) (p< 0.05). Lymphocyte proliferation induced with PHA was enhanced in spleen cells from AG treated animals (D) (p<0.05).

iNOS blockade with AG has no effect on anti *N*. *brasiliensis* IgG and IgM antibody production in treated and infected mice (Fig 7A and 7B). In this group the IgM-PFC response to an unrelated antigen such as SRBC was not affected, as summarized in Fig 7C.

### Lymphocyte proliferation is enhanced with AG treatment

The splenocytes culture from AG treated and infected mice, showed a high stimulation index to PHA stimulation in contrast with the splenocytes of infected mice without treatment; in addition, it was found that AG itself enhances splenocytes proliferation presented in Fig 7D.

### AG treatment induces reduction of inflammatory cells in the infected tissue and inhibited spleen enlargement of infected mice

**Fig 8:**
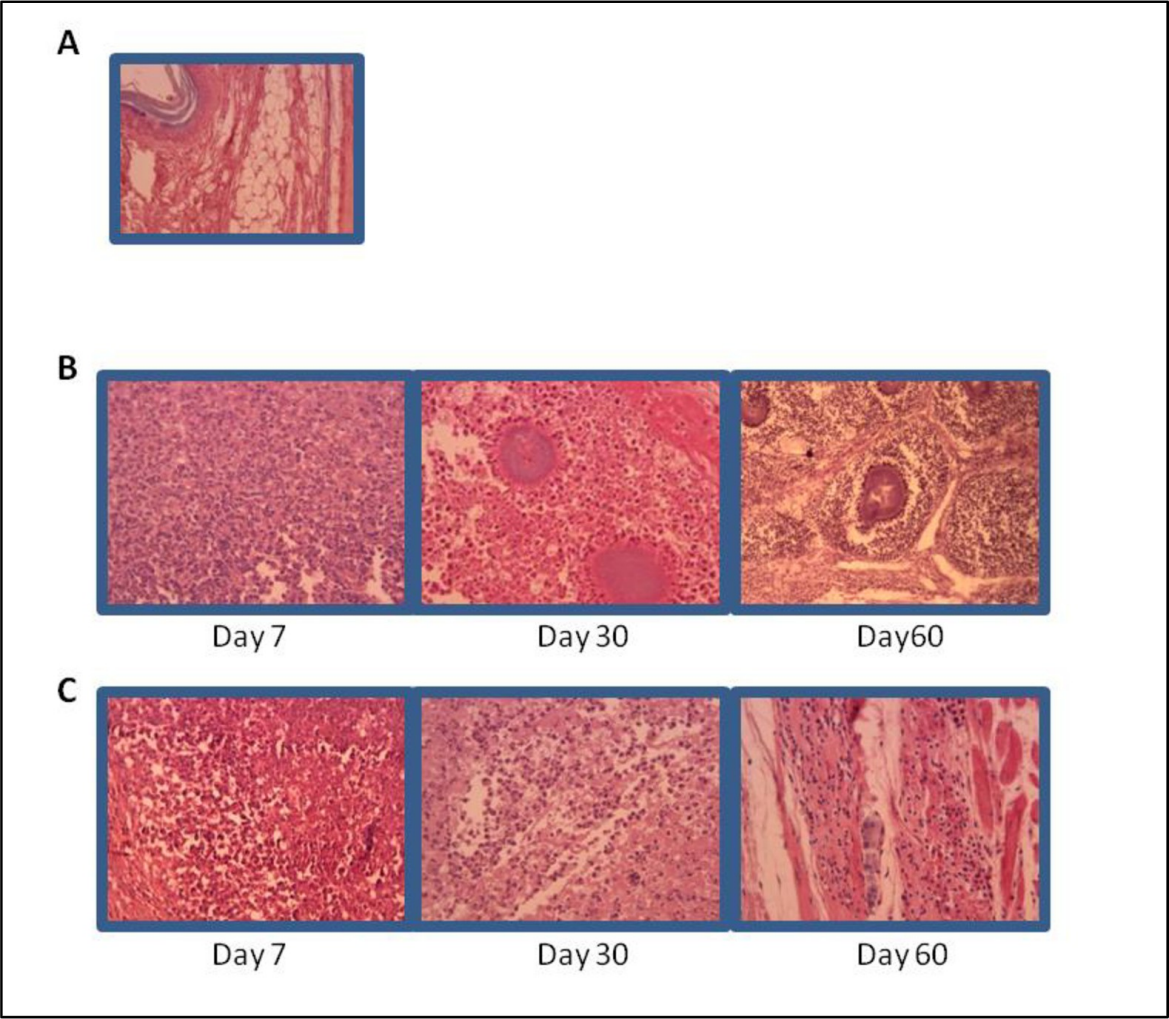
Inflammatory cell infiltration in infected tissue is decreased in the AG treated group. H&E. stained sections of BALB/c mice tissue at different times of the infection with *N*. *brasiliensis* A) Non treated non infected mice tissue. B) Non treated and infected group with microabsceses and micro colonies of *N*. *brasiliensis* C) AG treated and infected group showed a smaller number of inflammatory cells and no bacterial colonies.

**Fig 9:**
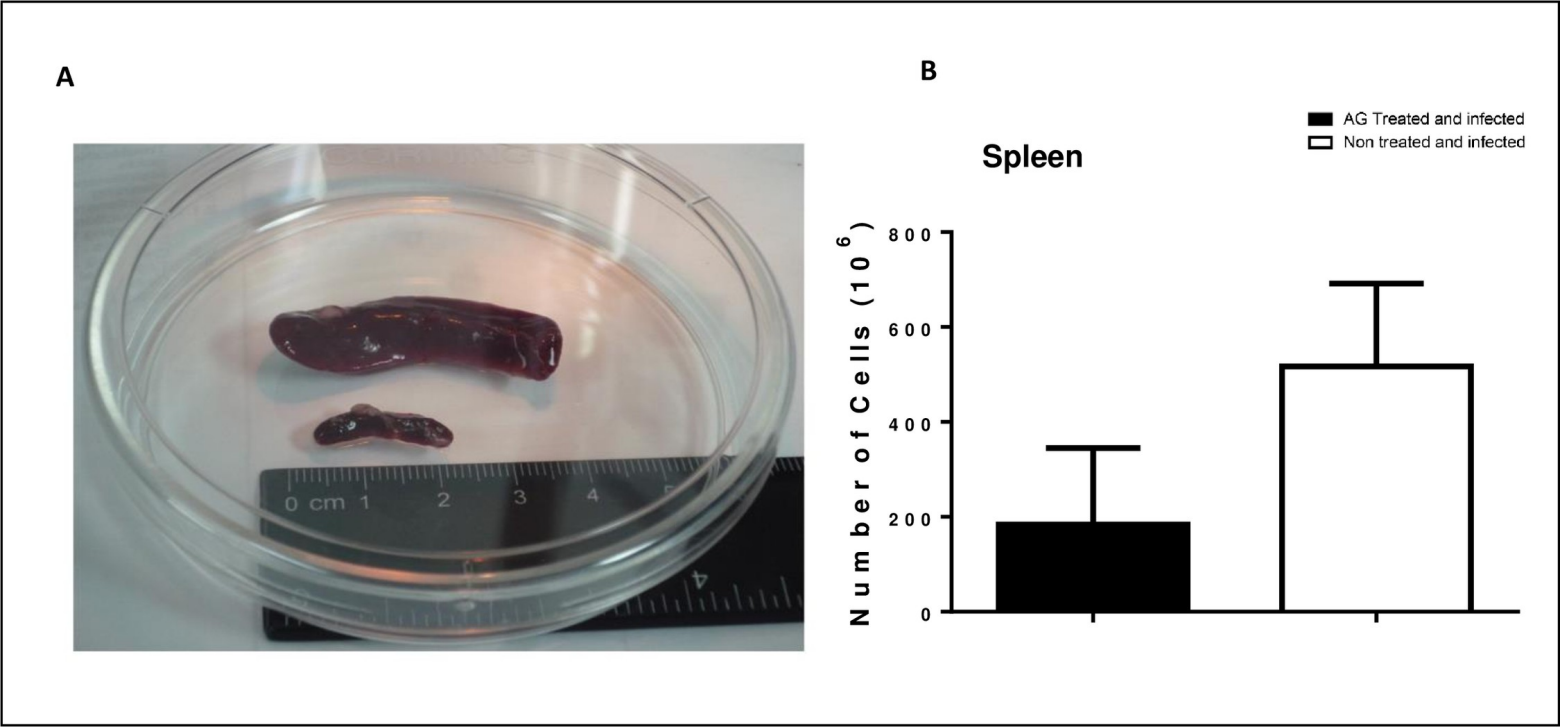
Spleen size and cell number obtained from non treated and infected mice was dramatically increased. A) Non treated and infected mice presented splenomegaly (top) compared to AG treated and infected animal (bottom). B) Splenocytes number from non treated and infected mice showed a statistically significant increase. (p<0.05).

Histopathological analysis of foot pads from AG treated animals, showed fewer number of inflammatory cells in the infected tissue, this finding was more evident at later days after infection, with an increased number of inflammatory cells and microabsceses at days 30 and 60 as presented in Fig 8B. Spleen size from infected animals were measured at day 60 and showed that, it was 2 to 3 times larger in non treated and infected mice, compared to the AG treated non infected mice as in Fig 9A. The cell number recovered from spleen was dramatically increased in non treated and infected mice in comparison with AG treated and infected group as presented in Fig 9B.

### *Nocardia brasiliensis* bacterial load after AG treatment

*Nocardia brasiliensis* colony forming units (CFU) were absent in blood, kidney, liver and spleen cultures from both treated and non treated mice at the end of the experiment demonstrating that *N*. *brasiliensis* did not disseminate.

## Discussion

Mycetoma has been listed as a neglected tropical disease because it has been little addressed by scientist, governments and public health policy makers. Using the experimental mice model currently available, this infectious disease offers outstanding experimental opportunities to study both acute and chronic inflammation, granuloma formation, the biology of foamy cell formation, the pharmacological effect of new antibiotics, chemotherapies and vaccine development [20].

The pathogenesis of mycetoma in general, and in particular of actinomycetoma by *N*. *brasiliensis* is little known. Most information was obtained in a BALB/c mice model. Using this rodent model, it was reported that a hyper-immune sera protected mice from actinomycetoma by *N*. *brasiliensis* [6]. Later it was demonstrated that monoclonal IgM but not IgG anti-*N*. *brasiliensis* antibodies protects mice and prevent actinomycetoma development [7]. This information paved the way for a vaccine development to prevent this infection in endemic areas. These last two findings are in agreement with previous information demonstrating that humoral immunity has an important role in the protection of various intracellular microbes [5–8]. Controversy on the protective role of antibodies against intracellular bacteria can be explained in part, by a low or a very high concentration of protective antibodies in the hyper immune sera used in passive immunity trials. This fact could be responsible in part for the non-reproducible effect of similar protocols as discussed by Casadevall [8]. In the present study we found that AG treatment of mice infected with *N*. *brasiliensis* prevented actinomycetoma development but this protective effect is not due to antibody production. The specific and nonspecific antibody response remains unaffected by AG treatment, therefore, we can rule out the role of anti-*N*. *brasiliensis* antibodies in this protection. On the other hand, PHA-stimulated T lymphocyte proliferation was increased in the same AG treated animals. Whether this proliferative ability of T lymphocytes is related to protection is not conclusive with these results.

Nitric oxide synthase (NOS2) is the enzyme responsible for nitric oxide (NO) synthesis by macrophages when stimulated by interferons or microbial molecules as lipopolysaccharide (LPS). The role of NO in immunology has dramatically changed from a microbicide molecule to an inorganic radical that regulates innate and adaptive immunity [11]. We present herein experimental results that show that blocking the enzyme NOS2, with AG in mice infected with *N*. *brasiliensis*, protected 100% of mice from developing actinomycetoma. This surprising finding is not due to direct AG effect on *N*. *brasiliensis* since in vivo and ex vivo experiments demonstrate that viability, morphology or the ability to form colonies remains intact in in vitro bacterial cells cultured with high amounts of AG in vitro. Some deaths occurred in the AG treated and infected group but in a lower number than non treated and infected mice, we have no explanation for this finding, however, animals that received AG treatment and not infected, survived 100% so it is clear that AG is not responsible for deaths.

Inflammation, determined by volume in millimeters in macroscopic evaluation of infected tissue, and microscopic preparations, was unexpectedly dramatically reduced. Infected tissue examined in microscopic slides, also presented few numbers of inflammatory cells. The anti-inflammatory effect of AG treatment is not only local in infected tissue but rather systemic since spleen size and number of its cell content is also small compared to the infected non AG treated group. It is known that NOS2 is induced in macrophages and neutrophils by bacterial products, but it is also accepted that NOS2 is not present in resting cells. The decrease in inflammation may be explained in part because NO is produced by local inflammatory cells such as macrophages and polymorphonuclear cells which are absent in *N*. *brasiliensis* -infected tissue in AG treated animals. In addition, it has been demonstrated that NO participates in adaptive immunity by inhibiting T cell proliferation [21]. This supports our finding that T cell proliferation was increased in PHA stimulated lymphocytes in cells derived from AG treated animals. This result is also in line with our observation that the macrophage ability to kill *N*. *brasiliensis* cells was also decreased in cells obtained from naïve AG treated mice. This finding is clearly related to a reduction in NO production as found in our experiments and as published before [22]. The NO mechanism to increase T cell proliferation induced by mitogen stimulation as found in this work, also deserves further investigation not only in this actinomycetoma model but in other infectious diseases as well.

It has been published in some experimental models of infection that NO helps clear infection by *C*. *albicans* by increasing neutrophil phagocytosis [13]. Researchers findings using *Campylobacter jejuni* in an experimental infection agree with the beneficial effect of blocking NOS2 with AG [17]. However, using *Toxoplasma gondii*, other authors have reported that inducible NO is necessary to control the chronic phase but not acute infection [16].

Our findings and the results of other investigators need additional experiments to understand the role of NO in modulating the innate and adaptive immune response to different microbes. New information will be of help to pave the way for future medical interventions including vaccine development and new therapeutic targets.
